# Decannulation of tracheotomized patients after long-term mechanical ventilation – results of a prospective multicentric study in German neurological early rehabilitation hospitals

**DOI:** 10.1186/s12871-018-0527-3

**Published:** 2018-06-13

**Authors:** Maria-Dorothea Heidler, Annett Salzwedel, Michael Jöbges, Olaf Lück, Christian Dohle, Michael Seifert, Andrea von Helden, Wibke Hollweg, Heinz Völler

**Affiliations:** 1 University of Potsdam, Center of Rehabilitation Research, Am Neuen Palais 10, 14469 Potsdam, Germany; 2 Brandenburg Klinik Bernau, Bernau bei Berlin, Germany; 3 Kliniken Beelitz, Beelitz, Germany; 4 MEDIAN Klinik Berlin-Kladow, Berlin, Germany; 5 MEDIAN Klinik Grünheide, Grünheide (Mark), Germany; 6 Vivantes Klinikum Spandau, Berlin, Germany

**Keywords:** Mechanical ventilation, Tracheostomy, Decannulation, Prognosis

## Abstract

**Background:**

In the course of neurological early rehabilitation, decannulation is attempted in tracheotomized patients after weaning due to its considerable prognostic significance. We aimed to identify predictors of a successful tracheostomy decannulation.

**Methods:**

From 09/2014 to 03/2016, 831 tracheotomized and weaned patients (65.4 ± 12.9 years, 68% male) were included consecutively in a prospective multicentric observation study. At admission, sociodemographic and clinical data (e.g. relevant neurological and internistic diseases, duration of mechanical ventilation, tracheotomy technique, and nutrition) as well as functional assessments (Coma Recovery Scale-Revised (CRS-R), Early Rehabilitation Barthel Index, Bogenhausener Dysphagia Score) were collected. Complications and the success of the decannulation procedure were documented at discharge.

**Results:**

Four hundred seventy patients (57%) were decannulated. The probability of decannulation was significantly negatively associated with increasing age (OR 0.68 per SD = 12.9 years, *p* < 0.001), prolonged duration of mechanical ventilation (OR 0.57 per 33.2 days, p < 0.001) and complications. An oral diet (OR 3.80; *p* < 0.001) and a higher alertness at admission (OR 3.07 per 7.18 CRS-R points; *p* < 0.001) were positively associated.

**Conclusions:**

This study identified practically measurable predictors of decannulation, which in the future can be used for a decannulation prognosis and supply optimization at admission in the neurological early rehabilitation clinic.

## Background

The number of tracheotomies conducted for more comfortable long-term ventilation has increased rapidly in recent years. Therefore, a growing number of patients with a tracheal cannula (TC) will be transferred from Intensive Care Units (ICU) to neurological-neurosurgery early rehabilitation clinics (ERC) to be weaned (ERC are intermediate units for the rehabilitation of severely care-dependent patients in Germany). A study on the long-term outcome of early rehabilitation patients showed that this weaning is of outstanding importance. If it fails, the probability of survival is significantly reduced after discharge from the ERC: One year later, less than 50% of the patients who were discharged with a TC survived [1]. Therefore, for a therapeutic and economical optimization of decannulation management, it is of great interest which factors make the success or failure of decannulation more likely.

In recent years, the attempt has often been made to identify suitable decannulation predictors. These include, for example, a Peak Cough Flow > 160 l/min [2], a sufficiently high Cough Peak Flow Rate in induced cough [3] or a sufficient peripheral and respiratory muscular strength [4]. A systematic review [5] showed that effective coughing and tolerance of TC occlusion ≧ 24 h were the most relevant parameters for decannulation in clinical practice. In addition, the degree of consciousness, the condition of the tracheal secretion, the swallowing capacity, the respiratory stability before and after TC occlusion (paCO2 < 60 mmHg), bronchoscopically secured tracheal stenosis, the indications for tracheotomy and the number of comorbidities (≥ 1) proved to be important predictive parameters.

A problem with these predictors is, above all, how to ascertain them. On the one hand, measurements (e.g. the Cough Peak Flow Rate) require specific measuring instruments which are not usually available in the ERC. On the other hand, many factors are difficult to quantify (e.g., a “sufficiently high” muscular strength) or can be distorted by a subjective bias (e.g., the assessment of swallowing ability or secretion). The aim of this study was therefore to identify practicable predictors for successful decannulation in the ERC.

## Methods

### Sample and data collection

In a prospective multicentric registry study, the medical routine data of *n* = 831 consecutive tracheotomized ERC patients were collected. A patient consent was not required, because only routine medical data were collected and evaluated anonymously and no interventions were performed. The routine data were documented at the participating clinics without personal data (name, place of residence, etc.)**.** The recruiting was carried out between 09/2014 and 03/2016 at five ERCs in the Berlin/Brandenburg area. Inclusion criteria were a tracheotomy due to invasive mechanical long-term ventilation and a successful weaning from mechanical ventilation in the ICU or ERC.

We collected sociodemographic data (age, sex), medical data (type of critical illness, neurological, cardiac, pulmonary, renal, gastrointestinal, oncological, orthopedic and psychiatric comorbidities, respiratory parameters, tracheotomy technique), functional assessments (Early Rehabilitation Barthel Index, Bogenhausener Dysphagia Score, Coma Recovery Scale-Revised), and complications during the decannulation and rehabilitation periods (pneumonia, sepsis, laryngeal edema, tracheal stenosis, tracheomalacia). The primary endpoint was the decannulation status at discharge from the ERC (decannulated vs. nondecannulated). A decannulation was being assessed as successful if no respiratory complications occurred during the patient‘s stay in the ERC (or at least 2 weeks after decannulation). A standardized procedure for tracheal cannula management [6] was not available for all five participating clinics.

### Statistical analysis

Continuous variables are represented as mean ± standard deviation, categorial variables as absolute values and percentages. Group differences were determined by t-test or Chi²-test. The primary endpoint was analyzed using a binary logistic regression model with stepwise backwards elimination. The following covariates were taken into account: age at admission, sex, CRS-R at admission, number of complications, duration of mechanical ventilation, chronic neurological disease, acute cerebral infarction, acute nontraumatic hemorrhage, acute traumatic brain injury, acute hypoxic brain damage, Morbus Parkinson, acute critical illness polyneuro−/myopathy (CIP/CIM), acute epilepsy, cardiac disease (no vs. acute vs. only chronic), pulmonary disease, gastrointestinal, oncological, renal, orthopedic and psychiatric disorders (no vs. acute vs. chronic), addictive disorder, sepsis, obesity (BMI > 30), diabetes mellitus, type of tracheotomy (dilatational vs. surgical) and complications (none vs. pneumonia or respiratory infections vs. other infections or sepsis or other). Patients who died with a TC remained in the model. A statistical significance was assumed for effects with a *p*-value < 0.05.

## Results

The investigated patients had a high degree of multimorbidity: In addition to neurological diseases, 90.6% of the patients had pulmonary, 64% cardiac, 39.1% psychiatric, 30% renal and 24.9% gastrointestinal acute or chronic comorbidities.

Of the 831 patients, 470 were successfully decannulated (57%). These were, on average, younger (64 vs. 67 years, *p* < 0.001) and had fewer common cerebral infarctions (*p* = 0.003), less hypoxic brain damage (*p* = 0.014), fewer epileptic seizures (*p* < 0.001) and fewer cardiac (*p* = 0.010) or pulmonary diseases (*p* = 0.018) than patients who could not be decannulated (Table 1). In addition, decannulated patients had significantly higher scores for CRS-R (19.3 ± 5.2) than nondecannulated (12.9 ± 7.7) at admission to the ERC, and were more frequently orally fed (28.7%) compared to only 4.4% of the nondecannulated patients.

**Table 1:** Patient characteristics at admission to ERC (*n* = 831)

Parameters at admission	Total (*n* = 831)	Decannulated (*n* = 470)	Non-decannulated (*n* = 361)	*p*-value
	M ± SD or n (%)	M ± SD or n (%)	M ± SD or n (%)	
Age (years)	65.4 ± 12.9	63.9 ± 12.8	67.3 ± 12.8	< 0.001
Sex (male)	565 (68.0)	303 (64.5)	262 (72.6)	0.013
Cerebral infarction	243 (29.2)	118 (25.1)	125 (34.6)	0.003
Nontraumatic hemorrhage	210 (25.3)	114 (24.3)	96 (26.6)	0.442
Traumatic brain injury	116 (14.0)	59 (12.6)	57 (15.8)	0.182
Hypoxia	117 (14.1)	54 (11.5)	63 (17.5)	0.014
CIP/CIM	313 (37.7)	196 (41.7)	117 (32.4)	0.006
Epilepsy	137 (16.5)	59 (12.6)	78 (21.6)	< 0.001
Cardiac disease	532 (64.0)	294 (62.6)	23 (65.9)	0.315
Acute or acute-chronic	302 (36,3)	183 (38,9)	119 (33.0)	
Chronic	232 (27.9)	112 (23.8)	120 (33.2)	
Pulmonary disease	753 (90.6)	416 (88.5)	337 (93.4)	0.018
Acute or acute-chronic	728 (87.6)	402 (85.5)	326 (90.3)	0.060
Chronic	25 (3.0)	14 (3.0)	11 (3.0)	
Gastrointestinal disease	207 (24.9)	116 (24.7)	91 (25.2)	0,862
Acute or acute-chronic	152 (18.3)	87 (18.5)	65 (18.0)	0.833
Chronic	55 (6.6)	29 (6.2)	26 (7.2)	
Renal disease	249 (30.0)	143 (30.4)	106 (29.4)	0.740
Acute or acute-chronic	120 (14.4)	71 (15.1)	49 (13.6)	0.821
Chronic	129 (15.5)	72 (15.3)	57 (15.8)	
Psychiatric disease	325 (39.1)	204 (43.4)	121 (335)	0.001
Addictive disorder	213 (25.6)	139 (29.6)	74 (20.5)	0.010
Sepsis	313 (37.7)	174 (37.0)	139 (38.5)	0.662
Tracheotomy technique				< 0.001
Dilatational	435 (52.3)	287 (61.1)	148 (41.0)	
Surgical	396 (47.7)	183 (38.9)	213 (59,0)	
Nutrition				< 0.001
Nasogastric tube	346 (41.6)	194 (41.3)	152 (42.1)	
PEG	334 (40.2)	141 (30.0)	193 (53.5)	
Oral diet	151 (18.2)	135 (28.7)	16 (4.4)	
CRS-R	16.6 ± 7.2	19.3 ± 5.2	12.9 ± 7.7	< 0.001

Results as mean ± standard deviation or number of cases (in percent)

The patients were ventilated an average of 48.8 ± 33.2 days. The duration of ventilation was significantly longer (*p* < 0.001) for nondecannulated patients. In addition, nondecannulated patients were more frequently affected by complications during the rehabilitation period, especially by pneumonia and respiratory infections. One-third of the patients (32.3%) were discharged from the ERC for further rehabilitation, another third (31.3%) into a nursing home and 10.8% back to the family. 12.3% of the patients had to be transferred back to the ICU. Sixty two patients (7.5%), 58 of them in the nondecannulated group (16.1%), died in the EFC (Table 2).

**Table 2:** Course of disease and outcome of ERC patients (*n* = 831)

Parameters at discharge	Total (*n* = 831)	Decannulated (*n* = 470)	Non-decannulated (*n* = 361)	*p*-value
	M ± SD or n (%)	M ± SD or n (%)	M ± SD or n (%)	
Wearing time of TC (days)	–	64.6 ± 36.0	–	
Duration of ventilation (days)	48.8 ± 33.2	43.6 ± 24.9	55.7 ± 40.6	< 0.001
Duration of stay in the ERC (days)	64.2 ± 48.1	63.3 ± 47.2	65.4 ± 49.3	0.547
Complications (existing)	283 (33.9)	102 (21.7)	181 (49.9)	< 0.001
Number of complications	0.5 ± 0.8	0.3 ± 0.6	0.7 ± 0.9	< 0.001
Complications (type)				< 0.001
None	550 (66.2)	368 (78.3)	182 (50.4)	
Pneumonia / respiratory infect	130 (15.6)	47 (10.0)	83 (23.0)	
Other infect / sepsis	64 (7.7)	27 (5.7)	37 (10.2)	
Other	87 (10.4)	28 (5.9)	59 (16.3)	
Nutrition				< 0.001
Nasogastric tube	28 (3.6)	1 (0.2)	27 (8.9)	
PEG	295 (38.4)	56 (12.0)	239 (78.9)	
Oral diet	446 (58.0)	409 (87.8)	37 (12.2)	
CRS-R	19.1 ± 6.3	21.9 ± 2.8	14.6 ± 7.6	< 0.001
Recannulation	24 (4.9)	8 (1.7)	16 (84.2)	< 0.001
Deceased	62 (7.5)	4 (0.9)	58 (16.1)	< 0.001
Status at discharge				< 0.001
Domesticity	90 (10.8)	70 (14.9)	20 (5.5)	
Further rehabilitation	268 (32.3)	264 (56.2)	4 (1.1)	
Nursing home	260 (31.3)	87 (18.5)	173 (47.9)	
Relocation to the ICU	102 (12.3)	31 (6.6)	71 (19.7)	
Other	49 (5.9)	14 (3.0)	35 (9.7)	
Death	62 (7.5)	4 (0.9)	58 (16.1)	

Results as mean ± standard deviation or number of cases (in percent)

**Fig. 1:**
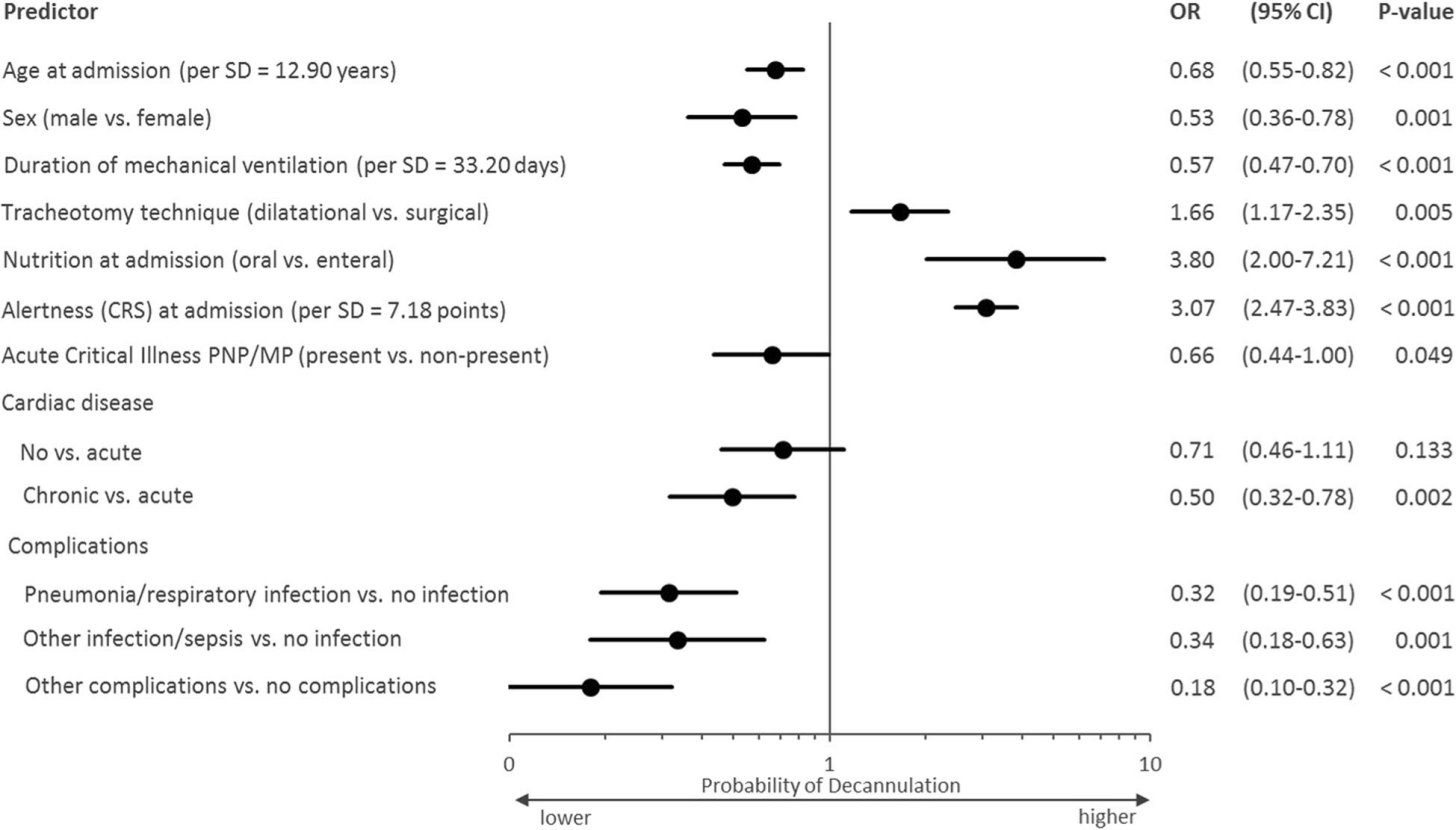
Multicentrically assessed predictors of decannulation (*n* = 831; Nagelkerkes R^2^ = 0,465). SD = standard deviation; OR = odds ratio; CI = confidence interval; PNP/MP = polyneuro−/myopathy

The likelihood of a successful decannulation was significantly reduced with increasing age (OR 0.68 per SD = 12.9 years, *p* < 0.001), a longer duration of mechanical ventilation (OR 0.57 per 33.2 days, *p* < 0.001) and complications, whereas an oral diet (OR 3.80; *p* < 0.001) and a higher responsivity at admission (OR 3.07 per 7.18 CRS-R points; *p* < 0.001) were positively associated (Fig.1).

## Discussion

In the present study, a large, tracheostomized patient collective in ERCs was investigated multicentrically for the first time. In addition to neurological diseases, these patients also had a wide range of internal medicine related diseases; independently, more than half of the patients could be weaned from the TC. Besides proving a high decannulation rate, it was also possible to specify predictors which could be easily assessed. Not only age and sex, but also the tracheotomy technique, as well as the level of alertness or responsivity and an oral diet at admission to the ERC were prognostically important. Complications during early rehabilitation had a limiting effect.

### Age and sex

While age in other studies [5, 7] had an effect on decannulation (younger patients had a higher probability of decannulation than older ones), the negative association with the male sex was rather unexpected. For example, a recent review of sex differences after stroke [8] shows a better functional outcome for male patients, as does a recent study [9] in geriatric patients after stroke (*n* = 919, 56% male), from which at least 55% of our patients suffered. However, nearly 70% of all patients in our study were male, which could have led to a bias.

### Cardiac diseases

Heart diseases also had an effect, whereby patients had a higher decannulation rate when cardiac disease was acute compared to chronic. One possible explanation for this could be the high prevalence of cognitive impairments that have an influence on voluntary secretion management and food intake (e.g., in terms of attentional focus or executive planning). For example, 50% of the patients experienced postoperative delirium after bypass surgery [10] and 25–74% of the chronic cardiac patients were cognitively impaired [11].

### Technique of tracheotomy

Patients with a dilatational tracheotomy had a 66% higher probability of decannulation than patients with a surgical tracheotomy. Numerous studies support our finding by showing a lower complication rate (e.g. fewer postoperative infections or less peristomal bleeding) for dilatational tracheotomy [12–15]. In addition, long-term complications such as tracheal stenosis are also less frequent after dilatational tracheotomy [16, 17]. On which basis the specific tracheotomy technique was chosen in the ICU cannot be assessed. Our data show a clear superiority of the dilatation tracheotomy with respect to decannulation.

### Complications

A high predictive value for decannulation had complications during the rehabilitation period. These included pneumonia and respiratory infections, which in tracheotomized patients can be caused by aspiration [18], and bacterial colonization after tracheotomy [19], but also by the TC itself [20]. Other complications, such as laryngeal edema, tracheomalacia, or tracheal stenosis, were astonishingly rare in the patients observed here compared to other studies [21], but did have an effect on decannulation.

### Duration of mechanical ventilation

In other studies, the duration of mechanical ventilation also had a negative effect on decannulation and on the patient‘s functional status at discharge [22]. With regard to weaning from the TC, we assume above all a negative influence on swallowing functions. Even a prolonged endotracheal intubation (≥ 48 h) is an independent predictor of dysphagia [23, 24] and leads to severe and persistent dysphagia with aspiration, independently of the underlying critical illness [25]. Also, the cuffed TC itself has a negative impact on deglutition: Above all, the absence of a physiological airflow through larynx, pharynx, nose and mouth is problematic because it is an important stimulus for spontaneous swallowing. Furthermore, the TC leads to a ubiquitous sensory impairment due to a lack of stimulation of chemo- and pressure receptors in the laryngeal mucosa. The longer the physiological airflow is interrupted by invasive mechanical ventilation, the more seriously deglutition processes can be impaired, which in turn requires a cuffed TC to protect the lower airways from aspiration [26]. Decannulation is therefore highly dependent on the extent to which swallowing functions (through physiological airflow control and dysphagia therapy) are improved.

### Nutrition / dysphagia

The type of feeding at admission to the ERC (through nasogastric tube, PEG or orally) had a significant influence on decannulation. Patients who had already had a PEG at admission could not be decannulated to a large extent compared to patients who took an oral diet. In this connection, the type of diet reflects the severity of swallowing. Dysphagia may be the result of neurological damage in areas relevant for swallowing or of the cuffed TC itself [27]. Since all invasively mechanically ventilated patients are at high risk for the development of dysphagia with aspiration, an exhaustive instrument-based or clinical swallowing test should be performed prior to oral feeding [26]; however, the decision criteria for the onset of an oral diet in the ICU were not collected, so that it cannot be ruled out that among the patients with oral diet at admission to the ERC were some with severe dysphagia.

### Alertness

A further predictor was the level of alertness / responsivity at admission to the ERC, which was assessed by CRS-R [28]. Decannulated patients had a significantly higher scale value at admission than nondecannulated patients. Presumably, alertness has a direct impact on other functions, such as safe food intake or effective secretion management. In patients with traumatic brain injury (*n* = 20, 80% male, age group 21–85), a close relationship was found between the level of consciousness and the probability of decannulation. A reduced awareness was associated with dysphagia, aspiration and pneumonia [27]. However, a significant influence could not be shown in all studies [3]. Moreover, “alertness” is difficult to operationalize. In assessments such as the CRS-R, parameters like attention or object recognition are quantified quantitatively by means of verbal and motor responses, which can be limited by peripheral or central paresis, disorientation, delirium or reduced instructional comprehension in critically ill patients. It is possible that the CRS-R parameter is an indicator of the severity of the cerebral disease per se, which limits consciousness and all levels of function.

### Critical illness polyneuro−/myopathy (CIP / CIM)

CIP and CIM are frequent complications in critically ill patients and affect the motor and sensory axons of the peripheral nervous system. An important risk factor is sepsis [29], which occurred in 37.7% of the patients examined here. A current electrophysiological study [30] on the frequency of CIP / CIM in early rehabilitation patients (*n* = 782) was able to demonstrate it in almost 70% of cases; the ventilation duration was significantly increased (*p* < 0.001), with an average of 32.1 days vs. 20.6 days in patients without CIP / CIM. In accordance with that, in our study patients with CIP / CIM could be decannulated significantly less frequently than patients without. The severity of the CIP / CIM may also have had an influence (e.g., on coughing and swallowing), but this has not been assessed.

### Limitations

Our study has limitations. Thus, the severity of the neurological and other critical illnesses and comorbidities which could have an effect on decannulation with regard to the resulting dysphagia was not assessed [31]. Also, parameters from the ICU (e.g., ventilation pressure or criteria for the tracheotomy technique used or the start of oral diet) were not available, so that their influence on decannulation could not be determined. In addition, some variables that had an influence on the decannulation in other studies or were difficult to objectify were not assessed, such as recurrent vomiting [7], the effectiveness of coughing, tracheal secretion or the therapeutic approach for decannulation. Also, parameters that reflect motor function (strength, mobilization, ability to sit up, etc.) that are important for swallowing were not collected, as motor scores were not a part of the routine procedure at four of the five participating clinics. Since no follow-up was carried out after the patients were discharged from the ERC, it is impossible to say whether patients had to be recannulated after this period. The number of recannulations required during the observation period itself was very low, affecting only 24 patients (4.9%) of whom 16 could not be decannulated.

## Conclusions

In the present study, predictors of decannulation from medical routine data could be determined in a large number of patients. Many of these parameters are already available at admission to the ERC and provide evidence of successful decannulation very early. In particular, patients with an oral diet, dilatational tracheotomy or high responsivity have a favorable prognosis. On the other hand, complications that occur during early rehabilitation should be given great attention in order not to threaten the success of the decannulation. Potentially modifiable predictors (alertness / responsiveness, swallowing) should be the therapeutic focus of the NNFR. Prospectively, an investigation on the predictive value of the predictors would be desirable in a setting outside the EFC, e.g., in the case of patients who are permanently provided with a TC in the outpatient area.

## Abbreviations

CIP/CIM: Critical illness polyneuro−/myopathy
CRS-R: Coma Recovery Scale-Revised
ERC: Early rehabilitation clinic
ICU: Intensive Care Unit
TC: Tracheal cannula
